# Referral process to further evaluate poor sleep in breast cancer survivors

**DOI:** 10.1002/cam4.4578

**Published:** 2022-02-06

**Authors:** Julie L. Otte, Yelena Chernyak, Shelley A. Johns, Lea' Jackson, Kandice K. Ludwig, Jill Dodson, Shalini Manchanda, Elizabeth Bufink, Claire Draucker

**Affiliations:** ^1^ Indiana University School of Nursing Indianapolis IN USA; ^2^ Indiana University School of Medicine Indianapolis Indiana USA; ^3^ Division of General Internal Medicine and Geriatrics Indiana University School of Medicine and Center for Health Services Research, Regenstrief Institute, Inc. Indianapolis Indiana USA; ^4^ Virginia Mason Memorial Hospital Yakima Washington USA; ^5^ Indiana University Health Indianapolis Indiana USA; ^6^ Section of Pulmonary, Critical Care, Sleep and Occupational Medicine Indiana University School of Medicine Indianapolis Indiana USA

**Keywords:** breast cancer survivor, referral, sleep, symptom management, treatment, women

## Abstract

**Objective:**

Breast cancer survivors (BCS) are twice as likely to report symptoms of poor sleep as those without cancer. However, sleep disorders are under‐assessed and under‐treated among BCS. The purpose of this study was to determine the portion of BCS who completed referral visits to a sleep specialist and identify the acceptability, facilitators, and barriers to the screening and referral process.

**Methods:**

BCS, who reported having sleep problems, completed questionnaires to screen for symptoms suggestive of sleep disorders. Those with symptoms suggestive of sleep apnea, movement disorders, narcolepsy, insomnia syndrome, or circadian disorders, they were referred to a sleep medicine physician or behavioral sleep medicine psychologist. Two months after the referral, participants were interviewed about their perceptions of the acceptability, barriers, and facilitators to sleep screenings and referrals.

**Results:**

Of 34 BCS assessed for eligibility, 29 were eligible and had sleep problems. Only eight of 29 participants (27.6%) completed the sleep referral process. Most thought the screening and referral process was acceptable. However, BCS identified barriers to completing the referral visit, including time, not seeing the need for treatment, insurance/sick leave concerns, and distance/transportation.

**Conclusion:**

Adequate evaluation and treatment of sleep disorders in BCS are rare. Creative solutions to address barriers to timely sleep referrals are needed to reduce long‐term negative consequences of inadequate sleep.

## Graphical abstract

The purpose of this study was to conduct a qualitative‐descriptive study to determine the portion of breast cancer survivors (BCS) who completed referral visits to a sleep specialist and identify the acceptability, facilitators, and barriers to the screening and referral process. Of 34 BCS assessed for eligibility, 29 were eligible and had sleep problems. Only eight of 29 participants (27.6%) completed the sleep referral process. Adequate evaluation and treatment of sleep disorders in BCS are rare. Creative solutions to address barriers to timely sleep referrals are needed to reduce long‐term negative consequences of inadequate sleep.

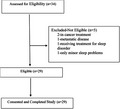

## 1 INTRODUCTION

Sleep insufficiency (lack of time asleep) and poor sleep quality are important public health problems that impact over 70 million persons in the United States and lead to an 11%–20% increase in health care costs.^1^ Sleep problems are associated with a variety of negative health outcomes, including poor health‐related quality of life, fatigue, poor healing, cognitive dysfunction, lost work productivity, accidents, poor relationships, and increased health care costs.^2^, ^3^, ^4^, ^5^, ^6^, ^7^, ^8^, ^9^, ^10^, ^11^, ^12^, ^13^ The costs of sleep insufficiency have been estimated to be over $107.5 billion.^1^ These costs and the human burden of reduced quality of life due to insufficient sleep far outweigh the costs incurred by appropriately assessing and adequately treating sleep problems.^1^

Breast cancer survivors (BCS) are among the many who are affected by poor sleep. Up to 90% of BCS report symptoms of poor sleep.^14^, ^15^, ^16^ Compared to women without cancer, BCS are twice as likely to report symptoms of poor sleep.^17^ Many BCS experience more than one sleep disorder (e.g., insomnia, sleep apnea, circadian rhythm disorders). The range of types of sleep disorders among BCS has not been fully reported until recently. Our research team conducted a separate study with a racially diverse sample of 38 BCS who reported sleep problems but had not been diagnosed with a sleep disorder.^16^ We screened participants for sleep disorders using a comprehensive, structured interview. Results showed that 97% of women had symptoms suggestive of insomnia disorder, 79% (*n* = 30) met minimum criteria for possible sleep apnea, 61% (*n* = 23) had symptoms of restless leg syndrome, 32% (*n* = 12) had symptoms suggestive of narcolepsy, and 84% (*n* = 32) had symptoms suggestive of a circadian rhythm disorder. In addition, 97% (*n* = 37) had symptoms suggestive of more than one disorder. African American participants had worse sleep quality and more symptoms suggestive of sleep apnea than Caucasian participants but a comparable number of possible sleep disorders. Participants were asked if they had ever discussed poor sleep with a provider or been accessed or referred for sleep assessment by a specialist. No participants endorsed these activities supporting previous research that oncology patients rarely discuss sleep problems with providers.^16^

Unfortunately, poor sleep continues to be one of the top five lingering symptoms reported by this population.^18^, ^19^, ^20^ For many BCS, poor sleep is experienced well into survivorship, with some reporting poor sleep for as many as 10 years post‐treatment.^14^ Although symptoms of poor sleep are common during survivorship, underlying sleep disorders are often not fully addressed by a provider.^21^ If left untreated, sleep problems can impact overall health and quality of life, leading to loss of work productivity, relationship problems, and a variety of comorbid conditions.^22^, ^23^

It is unclear why women's sleep problems receive inadequate attention during survivorship, given that significant strides have been made in the development of effective and accessible treatments for sleep disorders. Multiple studies with cancer patients have shown that cognitive behavioral therapy is as effective as a prescription medication for the long‐term treatment of insomnia.^24^, ^25^ Cognitive behavioral therapy for insomnia (CBT‐I) is the only treatment that has received ‘effective’ status by the Oncology Nursing Society due to strong evidence of its efficacy in cancer patients.^26^ Many behavioral treatments for insomnia can now be delivered effectively online or by telephone, individually or in a group setting..^27^, ^28^, ^29^ Although there are noted barriers in the literature regarding utilization of cognitive behavioral therapy for insomnia, the focus of this study was to first evaluate the referral process to gather preliminary data regarding referral uptake.^30^ This is a crucial step in understanding the behaviors related to treatment utilization.

Because chronic sleep problems are common, under‐assessed, and under‐treated in BCS, screening and referrals to sleep treatment are critical for this population. However, little is known about how best to implement these practices in cancer clinical settings. Understanding patients' perceptions of the acceptability, facilitators, and barriers to screening and referrals may help healthcare organizations determine how to address this population's sleep care needs. The purpose of this study was to examine BCS's with poor sleep experiences and reactions to the process of screening and referral to sleep treatment by a clinical provider. The specific aims were to (1) determine the portion of screened BCS who would complete a recommended referral visit with a sleep provider, (2) determine if BCS find the process of being screened and referred to a sleep provider acceptable, and (3) identify facilitators and barriers in the screening and referral process.

## 2 METHODS

A descriptive study using quantitative and qualitative data was conducted to address the study aims. Participants were BCS, who had completed surgery, radiation, and chemotherapy at least 6 months prior. The project was approved by the Indiana University–Purdue University Indianapolis (IUPUI) Institutional Review Board (IRB) and Indiana University Simon Cancer Center Scientific Review Committee. All participants provided informed consent and authorization for the use of their protected health information. Two trained research staff (one nurse, one social worker) obtained written consent from all participants.

### 2.1 Sample

Participants were recruited through convenience sampling from two oncology clinics in a major academic medical center. Female BCS were eligible if they met the following criteria^1^: At least 21 years of age^2^; willing and able to provide informed consent and human subjects authorization^3^; able to read, write, and speak English^4^; in good general health^5^; a non‐ metastatic breast cancer diagnosis (node involvement was acceptable)^6^; disease‐free for breast cancer at the time of study recruitment and enrollment^7^; at least 6 months post‐completion of surgery, radiation, chemotherapy, and/or herceptin therapy (endocrine therapy was allowed)^8^; report having sleep problems (inability to fall asleep, frequent awakenings, waking up too early, not feeling restful) for ≥two nights per week for the past 3 months,^9^ not currently being treated for a sleep disorder,^10^ no current major psychiatric disorder (e.g., schizophrenia, bipolar disorder),^11^ score > 7 on the Insomnia Severity Index (ISI) indicating high symptom burden of insomnia,^31^ and^12^ no current major or severe major depression, as determined by the Patient Health Questionnaire‐8 (PHQ‐8 score < 10).^32^ BCS with mild to moderate depression scores were eligible because these disorders are highly related to lack of sleep in this population. However, persons with severe depression were not due to safety concerns. If subjects scored in the severe range (PHQ‐8 > 10), they were informed of their score, and their oncologist was notified at the time of screening in the clinic. Recruitment took place over 7 months.

### 2.2 Procedures

The procedures used in this study followed a practice guideline for prevention, screening, assessment, and treatment of sleep disturbances in adults with cancer.^33^ First, the acting physician or nurse practitioner asked patients who were visiting the oncology clinic on a given recruitment day if they were experiencing poor sleep (yes or no). Recruitment occurred over a 7‐month period due to limited funding. If patients endorsed sleep problems and were interested in hearing about the study, they were referred to a research nurse within the clinic area for eligibility screening. Second, the research nurse screened patients to determine eligibility, which took on average between 5 and 10 min. BCS who were ineligible were thanked for their time and provided a brochure on healthy sleeping habits. BCS, who were eligible but not interested, were thanked for their time and queried by study personnel about why they were not interested in participating. Third, if eligible and interested, participants were consented, and the research nurse screened participants for symptoms suggestive of sleep disorder(s). Fourth, based on the screening results, participants were referred to a sleep specialist. If the screening revealed symptoms suggestive of sleep apnea, movement disorders, or narcolepsy, participants were referred to a board‐certified sleep medicine physician. If the screening revealed symptoms suggestive of insomnia syndrome disorders and/or circadian disorders, participants were referred to a behavioral sleep medicine psychologist. If participants needed a referral to both providers, the priority was given to the physician, as is standard practice. A member of the study team initiated the referral and contacted participants to schedule the initial appointment. The study team monitored this process to ensure appointments were scheduled for all participants. Fifth, participants were interviewed on the phone 2 months later to inquire about their experiences with the screening and referral process.

### 2.3 Measures

#### 2.3.1 Eligibility screening

A general inventory of demographic and health characteristics was used to determine eligibility. The research nurse administered the eligibility screening inventory, which included self‐reported, single‐item questions for eligibility criteria 1–10. For the remaining two criteria of sleep and depression, the ISI and PHQ‐8 were administered, respectively, both of which are standardized, well‐validated, and reliable instruments.

To establish the presence of poor sleep, the Insomnia Severity Index (ISI), a seven‐item questionnaire to detect the severity of perceived insomnia over the past 3 months, was used.^31^, ^34^ Response options (0 = *not at all* to 4 = *very much*) are summed for total scores ranging from 0 to 28. Higher scores indicate perceptions of greater insomnia severity. Reliability and validity have been established in healthy individuals (*n* = 145; Cronbach's alpha = 0.74) and cancer patients (*n* = 1634; Cronbach's alpha = 0.90).^31^, ^34^, ^35^ Scores greater than 7, which indicate subthreshold insomnia, were used for inclusion to allow persons with a wide range of severity of insomnia symptoms to participate.

To determine the absence of major and severe major depression, the Patient Health.

Questionnaire 8‐item depression scale (PHQ‐8) was used. The PHQ‐8 is a scale based on the Diagnostic and Statistical Manual of Mental Disorders, Fourth Edition (DSM‐IV) used to measure symptoms of major depressive disorder.^32^ Responses range from *not at all* = 0 to *nearly every day* = 3, resulting in a maximum summed score of 27. Higher scores represent increased depressive symptom severity. The PHQ‐8 is well‐validated and widely used as a brief diagnostic and severity measure of depression in cancer and non‐cancer populations.

#### 2.3.2 Demographic and health characteristics

A basic demographic questionnaire was completed by participants to obtain information about age, race, ethnicity, marital status, employment status, socio‐economic status, education, and menopausal status. Disease and treatment information was abstracted from the participants' medical records, including history of diabetes, thyroid disorder, hot flashes, arthritis, and depressive symptoms as well as years since breast cancer diagnosis, stage of disease, and type of treatment (surgery, chemotherapy, radiation, selective estrogen receptor modulators, and aromatase inhibitors).

#### 2.3.3 Screening for symptoms of sleep disorders

Research staff screened participants for symptoms suggestive of a sleep disorder that would need further evaluation by a sleep provider. Participants were informed that the initial screening would not diagnose a sleep disorder but rather determine if a referral to a sleep provider was indicated, and if so, to what type of sleep provider (physician or psychologist). A log was kept that chronicled to which sleep provider participants were referred and if participants completed the initial appointment. The following tools were used for screening.

#### 2.3.4 Pittsburgh Sleep Quality Index

The Pittsburgh Sleep Quality Index (PSQI) was designed for use in clinical populations as a simple and valid screening of sleep.^36^ The tool contains 19 items that produce a global sleep quality score based on seven component scores: sleep quality, sleep latency, sleep duration, habitual sleep efficiency, sleep disturbance, use of sleep medications, and daytime dysfunction. PSQI items use varying response categories, including Likert‐type responses. Responses are based on the prior month's habits. Scores of >5 are related to significantly poor sleep quality.^36^ Global sleep quality index scores >8 indicate symptoms of insomnia syndrome and related outcomes of daytime fatigue.^37^ Psychometrics such as content validity and internal consistency reliability have been widely supported in a variety of populations,^4^, ^37^, ^38^ including healthy individuals (*n* = 52; Cronbach's alpha = 0.83)^36^ and BCS (*n* = 102; Cronbach's alpha = 0.80).^37^

#### 2.3.5 STOP‐BANG (Snoring, tired, observed, pressure, body mass index, age, neck circumference, gender)

The STOP‐BANG is an 8‐item questionnaire used to determine the risk of sleep apnea. In previous studies, scores >3 had high sensitivity to predict moderate‐to‐severe (87.0%) and severe (70.4%) symptoms of sleep‐disordered breathing.^39^ Questions were administered by the trained research assistant. In addition, height/weight and body mass index were measured using a scale that has yearly calibration checks by institutional engineers, and neck circumference was measured using a standard paper tape measure. Each measure of height/weight and neck circumference was repeated twice for accuracy, and the average was used to calculate final values. Each STOP‐BANG question is weighted as a yes/no response, with 1 point for a yes and 0 for no.^39^, ^40^ Male gender is given 1 point in the questionnaire because research data suggests men have had a greater incidence of sleep apnea. However, the gender question was omitted for this study, given that all participants were female. Scores >3 prompted a referral to the sleep provider.

#### 2.3.6 Movement disorders

Single‐item questions were used to measure restless leg and periodic limb movements (parasomnias).^41^ Prior research has shown that these items are sensitive enough to guide referrals.^42^ Items were dichotomous (1 = yes or 0 = no). Scores >1 across the two items prompted a referral to the sleep provider.

#### 2.3.7 Epworth sleepiness scale

The daytime impact of sleep complaints was assessed using an 8‐item questionnaire of daytime sleepiness. The Epworth sleepiness scale (ESS) is one of the most common intake screening tools used in sleep medicine clinics. Reliability and validity have been established in adult populations (Cronbach's alpha = 0.88).^43^, ^44^ The ESS uses a numeric scale (0–3) to rate how likely someone is to fall asleep in eight different social or personal situations. Scores less than 10 are considered to be within the normal range. Scores >10 prompted a referral to the sleep provider.

#### 2.3.8 Circadian disorders

Since there are no brief tools available to screen for circadian disorders, items were selected from a standardized tool for initial screening. Seven items from the Sleep Disorders Questionnaire (version 2)^41^ were used to screen for signs of circadian disorders. The items were verified by the sleep medicine physician and co‐investigator, who agreed the items captured signs that would indicate a referral for further evaluation. Scores >4 prompted a referral to the sleep provider.

#### 2.3.9 Qualitative interviews

Two months following the referral, research staff conducted structured qualitative interviews with participants on a secure phone line. The two‐month timeframe was chosen as that is the typical wait time for university healthcare system appointments. Participants were asked whether they followed through with the referral, how they experienced the screening and referral process, and what facilitated and/or interfered with following through with the referral. The interviews were customized according to whether participants had been referred to a physician and/or psychologist. All interviews were audio‐ recorded and transcribed verbatim. Each interview was completed in 20–30 min.

### 2.4 Analysis

Quantitative data were entered into the Research Electronic Data Capture program^45^ servers located in a secure and environmentally structured computer operations center on the main campus of IUPUI. Research assistants verified data. Descriptive statistics were calculated for demographic, illness, and treatment variables.

Qualitative interview data were analyzed according to conventional content analytic procedures.^46^ All text units (e.g., relevant phrases or sentences) related to the study aims were extracted and coded with a short phrase that captured its essence. In addition to discussing their experiences with referrals, participants also discussed their experiences with sleep treatment, and these data were coded as well. A data display table^47^ was constructed with rows representing participants and columns representing one of four topics: acceptability of referral, barriers to referral, facilitators of referral, and experiences with sleep treatments. The participant rows were divided by whether the participants had completed the initial referral visit (e.g., completers versus non‐completers). Codes were placed in appropriate cells (e.g., Participant 001 × non‐completer × barriers to referral). Each column was then extracted as a separate data display table and similar codes were categorized. A narrative description of the categories of each column was prepared. Table 3, the data display table related to perceived barriers, is provided as an example of coding and categorization procedures.

Several procedures were used to ensure the rigor of the qualitative analysis. The research team met on several occasions to develop the analytic strategies and construct the initial data display table. The initial coding was conducted independently by two team members and the sets of codes were compared. Because the data were straightforward, there were few disagreements related to coding and these disagreements were easily resolved by a reexamination of the data. The categories were developed by team discussion and consensus. A qualitative methodologist who was not involved in the original coding of the data verified the codes, categories, and the narrative descriptions through review of the data. An audit trail that chronicled all analytic decisions was maintained.

## 3 RESULTS

### 3.1 Aim 1: Determine the portion of screened BCS who would complete a recommended referral visit with a sleep provider

**FIGURE 1:**
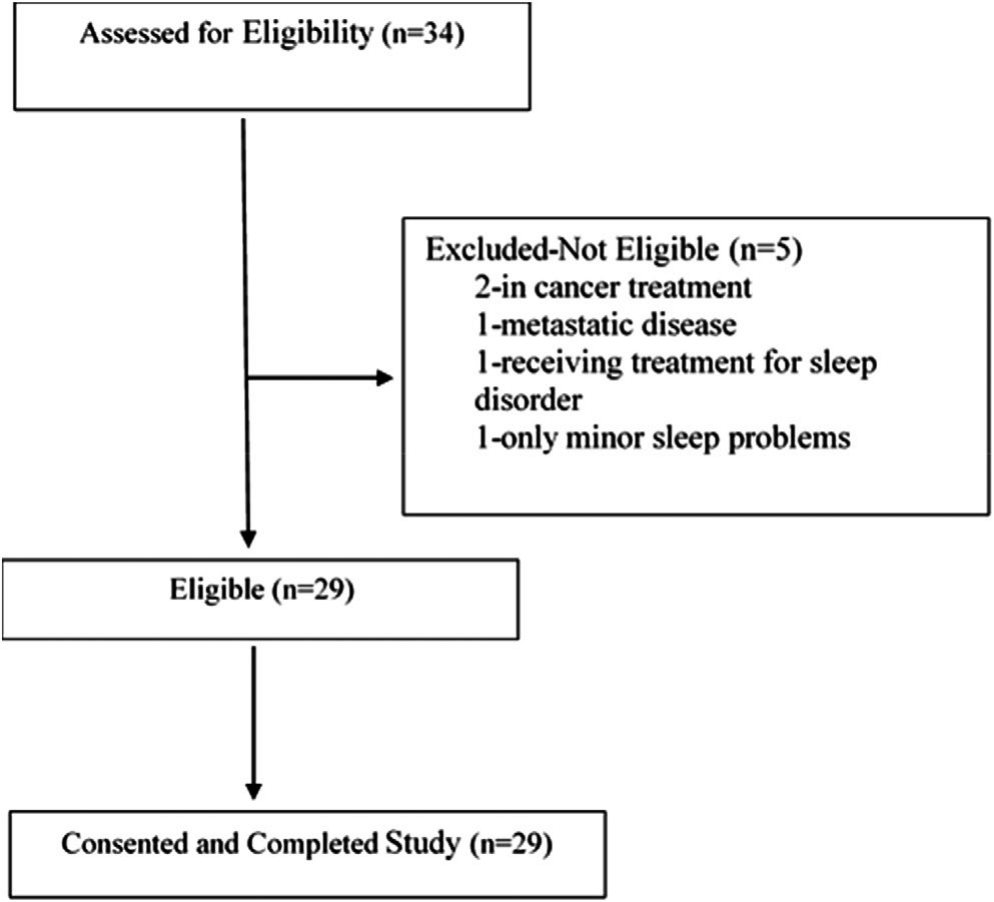
Study accrual flow diagram

Research staff screened 34 BCS for eligibility over a 7‐month period (Figure 1). Of the 34 screened, five were ineligible because they had minor sleep problems (*n* = 1), were undergoing treatment for cancer (*n* = 2), had metastatic disease (*n* = 1), or were actively receiving treatment for a sleep disorder (*n* = 1). The remaining 29 women were eligible, and all consented to participate in the study. Sample characteristics are presented in Table 1. Of these women, 26 screened high for insomnia severity, 23 screened positive for symptoms suggestive of circadian rhythm disorders, seven screened positive for symptoms of sleep apnea, six screened positive for possible movement disorders, and five screened positive for excessive daytime sleepiness. Sleep symptoms are reported in Table 2.

**TABLE 1:** Demographic characteristics

Patient characteristics	*N* = 29
	*M* (SD)
Age	59.69 (8.96)
BMI	28.97 (6.67)
Depressive symptoms (PHQ‐8 total score)	6.97 (4.14)
Years since diagnosis	7.97 (5.5)
	***N* (%)**
Ethnicity	
Hispanic	0 (0)
Race	
Black	9 (32.14)
White	19 (67.86)
Marital Status	
Married or partnered	15 (53.57)
Education	
High School degree	8 (27.59)
Undergraduate degree	13 (44.83)
Graduate degree	8 (27.59)
Employment	
Full or part‐time	15 (53.57)
Not employed or other	13 (46.43)
Difficulty for basic needs	
A lot of difficulty	3 (10.34)
Some difficulty	9 (31.03)
No difficulty	17 (10.34)
Postmenopausal	24 (82.76)
Diabetes	2 (6.90)
Thyroid disorder	5 (17.24)
Hot flashes	16 (55.17)
Arthritis	13 (44.83)
Stage of disease	
0–1	16 (55.17)
2–3	13 (44.83)
Type of treatment	
Surgery only	8 (27.59)
Surgery and radiotherapy	7 (24.14)
Surgery and chemotherapy	5 (17.24)
Surgery, radiotherapy, chemotherapy	9 (31.03)

**TABLE 2:** Major sleep outcomes

Sleep symptoms	*N* = 29
	*M* (SD)
Daytime sleepiness (ESS score)	5.93 (3.44)
Insomnia severity (ISI)	16.07 (5.18)
Global sleep quality (PSQI global)	12.72 (3.78)
Symptoms of sleep apnea (STOP‐BANG)	1.86 (1.25)
	***N* (%)**
Signs of restless leg and limb movement (yes/no)	9 (31%)
Signs of circadian rhythm disruptions (yes/no)	12 (41%)

Abbreviations: ESS, Epworth Sleepiness Scale; ISI, Insomnia Severity Index; PSQI, Pittsburgh Sleep Quality Index; STOP‐BANG, Snoring, Tired, Observed, Pressure, BMI, Age, Neck circumference, Gender.

**TABLE 3:** Example of data display table: Perceived barriers to sleep referral

Subject ID	Initial code	Categories
101	Lack of availability (work/time)	*Time*
102	Lack of availability (time)	Lack of availability (work/time)
104	Lack of availability (work/time)	Lack of availability (time)
107	Personal reasons	Lack of availability (work/time)
109	Personal family issues	Lack of availability (work/time)
110	Lack of availability (work/time)	Lack of availability (work/time)
111	No need for treatment	Lack of availability (time/family)
114	Long distance to clinic	Lack of availability (work/time)
116	Lack of availability (work/time)	Lack of availability (time)
	No sick time	Lack of availability (work/time)
117	Lack of availability (time/family)	*Other life demands*
	Personal health issues	Personal reasons
	Multiple priorities other than the referral	Personal family issues
		Personal health issues
		Multiple priorities
118	Lack of availability (work/time)	Multiple personal priorities of meeting basic needs
	Insurance concerns	
		*Not seeing need for treatment*
120	Weather issues with travel	
		No need for treatment
121	Insurance concerns‐out of pocket expenses	No need for treatment
		No need for treatment
123	Lack of availability (time)	No need for treatment
125	Lack of availability (work/time)	*Insurance/sick leave concerns*
201	Multiple personal priorities of meeting basic needs	No sick time
		Insurance concerns‐out of pocket expenses
202	No need for treatment	Insurance concerns
203	Personal family issues	
204	Missed opportunity but still has desire to complete	*Distance/transportation*
		Long distance to clinic
		Weather issues with travel
205	No need for treatment Lack of transportation	Lack of transportation
207	No need for treatment	

Based on the screening results, two participants were referred to the physician only, 13 were referred to the behavioral sleep medicine psychologist only, and 14 had symptoms that suggested the need for both the physician and psychologist. Eight (27.6%) participants completed a referral visit, all of which were to the behavioral sleep medicine psychologist. The remaining 21 (72.4%) did not complete the referral visit; of these participants, two had been referred to the physician, 10 to the psychologist, and nine to both.

### 3.2 Aim 2: Determine if BCS find the process of being screened and referred to a sleep provider acceptable

Most of the participants who had completed an initial referral visit (hereafter referred to as completers) and those who did not (hereafter referred to as non‐completers) found the screening/referral process acceptable. No one objected to being screened. Only one participant, a completer, addressed what it was like more generally to be referred for sleep treatment. She remarked that the referral provided “a little extra validation that this [sleep] is an issue that can be treated successfully.”

### 3.3 Aim 3: Identify facilitators and barriers in the screening and referral process

With regard to facilitators of referral procedures, several remarked that the scheduler was helpful, the process ran smoothly, the initial visit was easy to schedule, and it was useful to have contact information “upfront.” They stated that they appreciated having study personnel contact the sleep provider as it circumvented the automatic scheduler that is typically used in the healthcare system. One participant stated, “They knew I was being referred so it all seemed very seamless.”.

While no participants identified barriers to being screened, many participants identified barriers to completing the initial referral visit or participating in ongoing treatment. Only one participant, a completer, identified a glitch related to the referral procedures used for the study. She cited problems setting up an initial appointment because the sleep provider to whom she was referred was on maternity leave, but this was resolved, and she did receive an appointment.

Another participant, a completer, remarked she had trouble scheduling ongoing appointments because the provider had limited availability. Except for one, no participants commented on clinic‐related barriers (e.g., a provider's limited availability). Instead, they commented on personal barriers. All participants who were non‐completers identified barriers to completing the referral related to their own personal circumstances rather than the referral procedures used for the study. Moreover, despite having completed at least one initial referral visit, some of the completers identified several of the same barriers that interfered with their ongoing sleep treatment. Given the relatively low referral visit completion rate and the extent of participants' concerns about barriers, the rest of this section is devoted to describing these barriers to referral and ongoing treatment. The main barriers that participants mentioned included time/other life demands, not viewing the need for sleep treatment, insurance/sick leave concerns, and distance/transportation challenges.

### 3.4 Time/other life demands

Nine non‐completers indicated that they did not follow through with the referral because of time constraints, especially related to their work schedules. For example, one participant said,

“I haven't followed through because of my work schedule. I thought I would be on nights by now, but I'm not. I'm still on days, and I don't get off until 6:00 pm, and you guys are closed.”

Five more non‐completers did not follow‐through because they were facing other more pressing life demands, including family illness and problems with their health. One participant said,

“I ended up not following through because my parents both became very ill and downward spiraled very quickly. We ended up putting my father in a nursing home and are still getting my mother around the clock care in home ‐ as well as my own personal battles right now – I am dealing with. Everything came at once, and this [sleep referral] just went down to the bottom of the list of priorities.”

One completer also revealed that she had begun to miss appointments because she experienced a number of illnesses that prevented her from following through with the sleep treatment.

### 3.5 Not seeing a need for treatment

Four non‐completers did not follow‐through with the referral because they did not feel they needed it. They remarked that their sleep problems had gotten better, they took sleep medicine that helped, or they considered their sleep problems to be normal or tolerable. One non‐completer stated, “I just felt my sleep patterns are kind of what they are, and I don't see that there is any underlying cause or anything.” Another non‐completer stated that she did not believe in psychology. Two of the completers who had been referred to both sleep providers followed through with just the psychologist but did not feel the need to see the physician because they felt the psychologist was helping them

### 3.6 Insurance/sick leave concerns

Three non‐completers had insurance and sick leave concerns that interfered with follow‐through. They were uncertain if the treatment was covered by insurance, did not want to use their insurance or their health savings account for this purpose, or did not have enough “banked” sick time. One stated,

“The way my insurance is set up, it would not have been beneficial for me to use my insurance for a study. I would rather save it for when things come up that I actually need help with.”

### 3.7 Distance/transportation

Three non‐completers did not follow‐through because they did not have transportation, the treatment site was too far away, or they were receiving treatment closer to home. One stated,

“I did not follow through simply because of where I am located. I am about a 3‐hour drive away, and winter around here is not conducive to long drives.”

One completer had no reliable transportation, so she discontinued her sleep treatment and sought help closer to home.

### 3.8 Experiences with sleep treatment

Although not directly related to the study aims of screening and referrals, we also gleaned some initial information regarding the experience of getting sleep treatment. The completers typically found the sleep treatment experience helpful. All described visits with the health psychologist to be good, very good, or informative. A few explained how they were asked to record their sleep patterns, change their sleep environment (e.g., make it dark), or meditate. Two indicated that their sleep had improved, and one felt it was “too soon to tell.”

## 4 DISCUSSION

To our knowledge, this study is the first to explore the experience of being screened and referred for sleep treatment from the perspectives of BCS. One of our primary findings was that the participants were not reluctant to be screened. Of the 29 eligible participants with sleep problems, all agreed to further sleep screening. This finding suggests that BCS are open to assessments related to their sleep. However, of the 29 participants referred to a sleep medicine provider, only eight followed through with the referral visit. Overall, this low rate of referral completion did not seem to be due to flaws in the referral procedures. Participants who commented on these procedures generally found them helpful, especially because they could avoid the automated scheduling system used by the health center. However, the replicability bypassing the automated call center for scheduling needs further investigation as it places additional burden on the healthcare team to make appointments.

The low rate of follow‐through with the referrals was mostly attributable to the women's personal circumstances related to other demands on their time, including work responsibilities and life stressors. This finding is consistent with other reports that indicate that women often neglect their sleep health and do not seek treatment for it even when poor sleep interrupts their daily functioning.^48^, ^49^, ^50^ Some experts suggest that women tend to see quality sleep as a luxury that is not prioritized because other responsibilities, such family caretaking, take precedence.^49^, ^51^ Our finding that some women did not see the need for sleep treatment because their sleep problems were “part of life” is consistent with other reports that BCS tend to normalize poor sleep as a common lifestyle problem.^49^

Our finding that some participants were concerned that sleep treatment was not covered by insurance or that they needed to save resources for other medical expenses that might come their way speaks to the concept of financial toxicity.^52^ Financial toxicity is the financial burden felt by patients and survivors that expands throughout the cancer trajectory.^52^ This concern may have been exacerbated because specialists were delivering the sleep treatment. Research has shown that 52% of cancer patients and survivors prefer to get their follow‐up care from their treating oncologists.^53^ The low rate of referral completion in our study might be due to participants' reluctance to engage with a new provider and the fear of additional expenses.

The findings of the study must be understood within the context of its limitations. The sample was small and represents only women who receive treatment and endorsed problems with sleep while being treated within an academic health system. The time frame of the 2‐month follow‐up was limited due to the short funding source therefore, future research needs to address longitudinal patterns of referral and treatment over time. Due to confidentiality, the total number of patients screened in this first step was not captured limiting the understanding on the full scope of poor sleep within the recruitment process. Most women had high levels of education and were financially stable and thus no conclusions can be drawn about women who are under‐resourced. The telephone follow‐up interviews were highly structured and more in‐depth, face‐to‐face interviews might provide additional information about how BCS experience sleep problems, how they view treatment for those sleep problems, and what they would recommend for healthcare providers who wish to create strategies to increase the rate of sleep treatment utilization in this population. Lastly, the replicability of patients having provider assistance with making referral appointments is limited as most larger healthcare systems use large call centers for appointments where patients have to make their own appointments.

Despite these limitations, the results suggest that providers should routinely screen BCS for sleep problems but be aware that the impediment to successful treatment utilization seems to occur when women are referred to a sleep specialist. Providers should thus attend to barriers at this juncture as perceived by BCS. Strategies can include educating BCS that improving the quality of their sleep is an important health goal rather than an indulgence and that untreated sleep disturbances are associated with a number of health risks. Providers can (1) initiate open conversations about how other life stressors might realistically interfere with follow‐through for sleep treatment, (2) address insurance and financial concerns related to sleep treatment, and (3) discuss the referral barriers for sleep referrals with sleep providers, such as location and transportation. Future research could also address the impact of patients having to use large call centers for appointments versus having assistance from a referral provider in making appointments to a sleep medicine specialist. It would be interesting to determine if making calls during the visit increases patient participation in those referral visits. BCS might also benefit from initial discussions about the variety of options, available to treat poor sleep.

## 5 CONCLUSIONS

This study provides new data regarding how BCS perceive the referral process for sleep problems that will be useful in planning future interventions. Future studies should aim to develop and test strategies that integrate sleep treatment in oncology or primary care, use health coaching with personalized messaging to increase sleep treatment utilization, offer a variety of evidence‐based treatment options, and investigate if there is potential stigma of psychological or behavioral care for sleep.

The results of this study show that sleep problems continue to be problematic in the long‐ term BCS at a level that needs further evaluation from a specialist. Without adequate treatment of sleep problems, BCS continue to be at risk for possible negative health outcomes, specifically poor health‐related quality of life, fatigue, poor healing, cognitive dysfunction, lost work productivity, safety issues (e.g., accidents), poor relationships, and increased health care costs. The knowledge generated from this study addresses several gaps on how cancer survivors perceive the process of seeking treatment for sleep problems that will better inform interventions focused on reducing the perceived and actual barriers that BCS experience to complete the referral to the sleep specialists. Additional research is needed to better evaluate the utilization of treatments once received. Creating pathways for BCS to prioritize their sleep health, receive ongoing sleep evaluations, and engage in treatments that are acceptable and effective could lead to improved health outcomes in this population.

## CONFLICT OF INTEREST

There are no conflicts of interest to disclose for any author listed on this manuscript.

## ETHICS APPROVAL AND CONSENT TO PARTICIPATE

The project was approved by the Indiana University–Purdue University Indianapolis (IUPUI) Institutional Review Board (IRB) and Indiana University Simon Cancer Center Scientific Review Committee. All participants provided informed consent and authorization for the use of their protected health information.

## Data Availability

Data Availability Statement: The data that support the findings of this study are available from the corresponding author upon reasonable request.

## References

[1] Centers for Disease Control and Prevention . Sleep and Sleep Disorders Atlanta: Centers for Disease Control and Prevention; 2014Available from: http://www.cdc.gov/sleep/index.html

[2] Ancoli‐Israel S , Moore PJ , Jones V . The relationship between fatigue and sleep in cancer patients: a review. Eur J Cancer Care. 2001;10(4):245‐255. 10.1046/j.1365-2354.2001.00263.x PMC2951731 11806675

[3] Anderson KO , Getto CJ , Mendoza TR , et al. Fatigue and sleep disturbance in patients with cancer, patients with clinical depression, and community‐dwelling adults. J Pain Symptom Manage. 2003;25(4):307‐318. 12691682 10.1016/s0885-3924(02)00682-6

[4] Beck SL , Schwartz AL , Towsley G , Dudley W , Barsevick A . Psychometric evaluation of the Pittsburgh sleep quality index in cancer patients. J Pain Symptom Manage. 2004;27(2):140‐148. 15157038 10.1016/j.jpainsymman.2003.12.002

[5] Berger AM , Higginbotham P . Correlates of fatigue during and following adjuvant breast cancer chemotherapy: a pilot study. Oncol Nurs Forum. 2000;27(9):1443‐1148. 11058976

[6] Bower JE , Ganz PA , Desmond KA , Rowland JH , Meyerowitz BE , Belin TR . Fatigue in breast cancer survivors: occurrence, correlates, and impact on quality of life. J Clin Oncol. 2000;18(4):743‐753. 10673515 10.1200/JCO.2000.18.4.743

[7] Carpenter JS , Elam J , Ridner S , Carney P , Cherry G , Cucullu H . Sleep, fatigue, and depressive symptoms in breast cancer survivors and matched healthy women experiencing hot flashes. Oncol Nurs Forum. 2004;31(3):591‐5598. 15146224 10.1188/04.onf.591-598

[8] Davidson JR , MacLean AW , Brundage MD , Schulze K . Sleep disturbance in cancer patients. Soc Sci Med. 2002;54(9):1309‐1321. 12058848 10.1016/s0277-9536(01)00043-0

[9] Dow KH , Ferrell BR , Leigh S , Ly J , Gulasekaram P . An evaluation of the quality of life among long‐term survivors of breast cancer. Breast Cancer Res Treat. 1996;39(3):261‐273. 8877006 10.1007/BF01806154

[10] Engstrom CA , Strohl RA , Rose L , Lewandowski L , Stefanek ME . Sleep alterations in cancer patients. Cancer Nurs. 1999;22(2):143‐148. 10217030 10.1097/00002820-199904000-00006

[11] Fortner BV , Stepanski EJ , Wang SC , Kasprowicz S , Durrence HH . Sleep and quality of life in breast cancer patients. J Pain Symptom Manage. 2002;24(5):471‐480. 12547047 10.1016/s0885-3924(02)00500-6

[12] Koopman C , Nouriani B , Erickson V , et al. Sleep disturbances in women with metastatic breast cancer. Breast J. 2002;8(6):362‐370. 12390359 10.1046/j.1524-4741.2002.08606.x

[13] Lee K , Cho M , Miaskowski C , Dodd M . Impaired sleep and rhythms in persons with cancer. Sleep Med Rev. 2004;8(3):199‐212. 15144962 10.1016/j.smrv.2003.10.001

[14] Otte JL , Carpenter JS , Russell KM , Bigatti S , Champion VL . Prevalence, severity, and correlates of sleep‐wake disturbances in long‐term breast cancer survivors. J Pain Symptom Manage. 2010;39(3):535‐547. 20083371 10.1016/j.jpainsymman.2009.07.004 PMC2843803

[15] Savard J , Simard S , Blanchet J , Ivers H , Morin CM . Prevalence, clinical characteristics, and risk factors for insomnia in the context of breast cancer. Sleep. 2001;24(5):583‐590. 11480655 10.1093/sleep/24.5.583

[16] Otte JL , Davis L , Carpenter JS , et al. Sleep disorders in breast cancer survivors. Support Care Cancer. 2016;24(10):4197‐4205. 27146391 10.1007/s00520-016-3247-6

[17] Jemal A , Bray F , Center MM , Ferlay J , Ward E , Forman D . Global cancer statistics. CA Cancer J Clin. 2011;61(2):69‐90. 21296855 10.3322/caac.20107

[18] Fox RS , Ancoli‐Israel S , Roesch SC , et al. Sleep disturbance and cancer‐related fatigue symptom cluster in breast cancer patients undergoing chemotherapy. Support Care Cancer. 2020;28(2):845‐855. 31161437 10.1007/s00520-019-04834-w PMC6959513

[19] Phillips KM , Jim HS , Donovan KA , Pinder‐Schenck MC , Jacobsen PB . Characteristics and correlates of sleep disturbances in cancer patients. Support Care Cancer. 2012;20(2):357‐365. 21311913 10.1007/s00520-011-1106-z

[20] Reynolds‐Cowie P , Fleming L . Living with persistent insomnia after cancer: a qualitative analysis of impact and management. Br J Health Psychol. 2021;26(1):33‐49. 32558129 10.1111/bjhp.12446

[21] Siefert ML , Hong F , Valcarce B , Berry DL . Patient and clinician communication of self‐reported insomnia during ambulatory cancer care clinic visits. Cancer Nurs. 2013;37:E51‐E59. 10.1097/NCC.0b013e318283a7bc PMC3696527 23448958

[22] Berger AM , Kumar G , LeVan TD , Meza JL . Symptom clusters and quality of life over 1 year in breast cancer patients receiving adjuvant chemotherapy. Asia Pac J Oncol Nurs. 2020;7(2):134‐140. 32478130 10.4103/apjon.apjon_57_19 PMC7233556

[23] Lai HL , Chen CI , Lu CY , Yao YC , Huang CY . Relationships among personality, coping, and concurrent health‐related quality of life in women with breast cancer. Breast Cancer. 2019;26(5):544‐551. 30747373 10.1007/s12282-019-00954-7

[24] Alshehri MM , Alenazi AM , Hoover JC , et al. Effect of cognitive behavioral therapy for insomnia on insomnia symptoms for individuals with type 2 diabetes: protocol for a pilot randomized controlled trial. JMIR Res Protoc. 2019;8(12):e14647. 31855189 10.2196/14647 PMC6940863

[25] Fiorentino L , McQuaid JR , Liu L , et al. Individual cognitive behavioral therapy for insomnia in breast cancer survivors: a randomized controlled crossover pilot study. Nat Sci Sleep. 2009;2010:1‐8. 10.2147/NSS.S8004 PMC2953254 23616695

[26] Matthews E , Carter P , Page M , Dean G , Berger A . Sleep‐wake disturbance: a systematic review of evidence‐based interventions for Management in Patients with Cancer. Clin J Oncol Nurs. 2018;22(1):37‐52. 10.1188/18.CJON.37-52 29350708

[27] Mann E , Smith M , Hellier J , Hunter MS . A randomised controlled trial of a cognitive behavioural intervention for women who have menopausal symptoms following breast cancer treatment (MENOS 1): trial protocol. BMC Cancer. 2011;11:44. 21281461 10.1186/1471-2407-11-44 PMC3045984

[28] Savard J , Simard S , Ivers H , Morin CM . Randomized study on the efficacy of cognitive‐behavioral therapy for insomnia secondary to breast cancer, part I: sleep and psychological effects. J Clin Oncol. 2005;23(25):6083‐6096. 16135475 10.1200/JCO.2005.09.548

[29] McCurry SM , Guthrie KA , Morin CM , et al. Telephone‐based cognitive behavioral therapy for insomnia in perimenopausal and postmenopausal women with vasomotor symptoms: a MsFLASH randomized clinical trial. JAMA Intern Med. 2016;176(7):913‐920. 27213646 10.1001/jamainternmed.2016.1795 PMC4935624

[30] Koffel E , Bramoweth AD , Ulmer CS . Increasing access to and utilization of cognitive behavioral therapy for insomnia (CBT‐I): a narrative review. J Gen Intern Med. 2018;33(6):955‐962. 29619651 10.1007/s11606-018-4390-1 PMC5975165

[31] Bastien CH , Vallieres A , Morin CM . Validation of the insomnia severity index as an outcome measure for insomnia research. Sleep Med. 2001;2(4):297‐307. 11438246 10.1016/s1389-9457(00)00065-4

[32] Kroenke K , Strine TW , Spitzer RL , Williams JB , Berry JT , Mokdad AH . The PHQ‐8 as a measure of current depression in the general population. J Affect Disord. 2009;114(1–3):163‐173. 18752852 10.1016/j.jad.2008.06.026

[33] Howell D , Oliver TK , Keller‐Olaman S , et al. A pan‐Canadian practice guideline: prevention, screening, assessment, and treatment of sleep disturbances in adults with cancer. Support Care Cancer. 2013;21(10):2695‐2706. 23708820 10.1007/s00520-013-1823-6

[34] Savard MH , Savard J , Simard S , Ivers H . Empirical validation of the insomnia severity index in cancer patients. Psycho‐Oncol. 2004;14(6):429‐441. 10.1002/pon.860 15376284

[35] Thorndike FP , Ritterband LM , Saylor DK , Magee JC , Gonder‐Frederick LA , Morin CM . Validation of the insomnia severity index as a web‐based measure. Behav Sleep Med. 2011;9(4):216‐223. 22003975 10.1080/15402002.2011.606766

[36] Buysse DJ , Reynolds CF 3rd , Monk TH , Berman SR , Kupfer DJ . The Pittsburgh sleep quality index: a new instrument for psychiatric practice and research. Psychiatry Res. 1989;28(2):193‐213. 2748771 10.1016/0165-1781(89)90047-4

[37] Carpenter JS , Andrykowski MA . Psychometric evaluation of the Pittsburgh sleep quality index. J Psychosom Res. 1998;45(1):5‐13. 9720850 10.1016/s0022-3999(97)00298-5

[38] Otte JL , Rand KL , Carpenter JS , Russell KM , Champion VL . Factor analysis of the Pittsburgh sleep quality index in breast cancer survivors. J Pain Symptom Manage. 2013;45(3):620‐627. 22926090 10.1016/j.jpainsymman.2012.03.008 PMC3535583

[39] Boynton G , Vahabzadeh A , Hammoud S , Ruzicka DL , Chervin RD . Validation of the STOP‐BANG questionnaire among patients referred for suspected obstructive sleep apnea. J Sleep Disord – Treat Care. 2013;2(4). 10.4172/2325-9639.1000121 PMC4008971 24800262

[40] Chung F , Yang Y , Liao P . Predictive performance of the STOP‐Bang score for identifying obstructive sleep apnea in obese patients. Obes Surg. 2013;23(12):2050‐2057. 23771818 10.1007/s11695-013-1006-z

[41] Douglass AB , Bornstein R , Nino‐Murcia G , et al. The sleep disorders questionnaire. I: creation and multivariate structure of SDQ. Sleep. 1994;17(2):160‐167. 8036370 10.1093/sleep/17.2.160

[42] Ferri R , Manconi M , Plazzi G , et al. Leg movements during wakefulness in restless legs syndrome: time structure and relationships with periodic leg movements during sleep. Sleep Med. 2012;13(5):529‐535. 22341907 10.1016/j.sleep.2011.08.007

[43] Johns MW . Reliability and factor analysis of the Epworth sleepiness scale. Sleep. 1992;15(4):376‐381. 1519015 10.1093/sleep/15.4.376

[44] Johns MW . A new method for measuring daytime sleepiness: the Epworth sleepiness scale. Sleep. 1991;14(6):540‐545. 1798888 10.1093/sleep/14.6.540

[45] Harris PA , Taylor R , Thielke R , Payne J , Gonzalez N , Conde JG . Research electronic data capture (REDCap)—a metadata‐driven methodology and workflow process for providing translational research informatics support. J Biomed Inform. 2009;42(2):377‐381. 18929686 10.1016/j.jbi.2008.08.010 PMC2700030

[46] Hsieh HF , Shannon SE . Three approaches to qualitative content analysis. Qual Health Res. 2005;15(9):1277‐1288. 16204405 10.1177/1049732305276687

[47] Miles MB , Huberman AM . Qualitative Data Analysis: An Expanded Sourcebook. Vol xiv. 2nd ed. Sage Publications; 1994:338.

[48] Cheek RE , Shaver JL , Lentz MJ . Lifestyle practices and nocturnal sleep in midlife women with and without insomnia. Biol Res Nurs. 2004;6(1):46‐58. 15186707 10.1177/1099800404263763

[49] Dzaja A , Arber S , Hislop J , et al. Women's sleep in health and disease. J Psychiatr Res. 2005;39(1):55‐76. 15504424 10.1016/j.jpsychires.2004.05.008

[50] Attarian HP , Schuman C . SpringerLink (Online Service). Clinical Handbook of Insomnia. Humana; 2010. 10.1007/978-1-60327-042-7

[51] Deater‐Deckard K , Chary M , McQuillan ME , Staples AD , Bates JE . Mothers' sleep deficits and cognitive performance: moderation by stress and age. PLoS One. 2021;16(1):e0241188. 33411778 10.1371/journal.pone.0241188 PMC7790244

[52] Lentz R , Benson AB 3rd , Kircher S . Financial toxicity in cancer care: prevalence, causes, consequences, and reduction strategies. J Surg Oncol. 2019;120(1):85‐92. 30650186 10.1002/jso.25374

[53] Hudson SV , Miller SM , Hemler J , et al. Adult cancer survivors discuss follow‐up in primary care: 'not what i want, but maybe what i need'. Ann Fam Med. 2012;10(5):418‐427. 22966105 10.1370/afm.1379 PMC3438209

